# Use of dose-area product to assess plan quality in robotic radiosurgery

**DOI:** 10.1016/j.zemedi.2023.01.001

**Published:** 2023-01-28

**Authors:** Markus Eichner, Alexandra Hellerbach, Mauritius Hoevels, Klaus Luyken, Michael Judge, Daniel Rueß, Maximilian Ruge, Martin Kocher, Stefan Hunsche, Harald Treuer

**Affiliations:** a Department of Stereotaxy and Functional Neurosurgery, Faculty of Medicine and University Hospital Cologne, University of Cologne, 50937 Cologne, Germany; b Department of Radiation Oncology, Cyberknife and Radiation Therapy, Faculty of Medicine and University Hospital Cologne, University of Cologne, 50937 Cologne, Germany

**Keywords:** Stereotactic radiosurgery, Dose-area product, Non-isocentric irradiation, Treatment plan optimization, Plan quality, Pareto efficiency

## Abstract

**Purpose:**

In robotic stereotactic radiosurgery (SRS), optimal selection of collimators from a set of fixed cones must be determined manually by trial and error. A unique and uniformly scaled metric to characterize plan quality could help identify Pareto-efficient treatment plans.

**Methods:**

The concept of dose-area product (DAP) was used to define a measure (DAP_ratio_) of the targeting efficiency of a set of beams by relating the integral DAP of the beams to the mean dose achieved in the target volume. In a retrospective study of five clinical cases of brain metastases with representative target volumes (range: 0.5–5.68 ml) and 121 treatment plans with all possible collimator choices, the DAP_ratio_ was determined along with other plan metrics (conformity index CI, gradient index R50%, treatment time, total number of monitor units TotalMU, radiotoxicity index f12, and energy efficiency index η50%), and the respective Spearman's rank correlation coefficients were calculated. The ability of DAP_ratio_ to determine Pareto efficiency for collimator selection at DAP_ratio_ < 1 and DAP_ratio_ < 0.9 was tested using scatter plots.

**Results:**

The DAP_ratio_ for all plans was on average 0.95 ± 0.13 (range: 0.61–1.31). Only the variance of the DAP_ratio_ was strongly dependent on the number of collimators. For each target, there was a strong or very strong correlation of DAP_ratio_ with all other metrics of plan quality. Only for R50% and η50% was there a moderate correlation with DAP_ratio_ for the plans of all targets combined, as R50% and η50% strongly depended on target size. Optimal treatment plans with CI, R50%, f12, and η50% close to 1 were clearly associated with DAP_ratio_ < 1, and plans with DAP_ratio_ < 0.9 were even superior, but at the cost of longer treatment times and higher total monitor units.

**Conclusions:**

The newly defined DAP_ratio_ has been demonstrated to be a metric that characterizes the target efficiency of a set of beams in robotic SRS in one single and uniformly scaled number. A DAP_ratio_ < 1 indicates Pareto efficiency. The trade-off between plan quality on the one hand and short treatment time or low total monitor units on the other hand is also represented by DAP_ratio_.

## 1 Introduction

Stereotactic radiosurgery (SRS) refers to a radiotherapy method used to treat benign or malignant brain pathologies with high doses delivered in only one or a few fractions to well-circumscribed targets [1]. In order to minimize normal tissue toxicity, SRS demands high conformity of the prescribed dose to the clinical target volume and rapid fall-off doses away from the target [2]. In robotic SRS this is typically achieved by use of inter- and intrafractional image guidance, by precise beam collimation using a set of cones and by application of non-isocentric beam arrays adapted to the target shape [3], [4], [5]. Inverse planning using the sequential [6] or VOLO^TM^ [7] optimization can be used to generate plans with high coverage and conformity while maintaining steep dose gradients and sparing of critical structures. While both algorithms can be used to determine appropriate beam weights, the selection of collimators from a set of fixed cones must be performed manually, which is time-consuming and generally has a large impact on plan quality [8]. There are well-established indices to quantify plan quality in terms of coverage, conformity, and dose gradient [1], [9], but currently there is no way to assess whether a plan is also Pareto-efficient with respect to collimator selection.

The aim of this study was to develop a method for determining optimal treatment plans in robotic SRS based on dose-area products (DAP) and to perform a Pareto efficiency analysis of the plans. Pareto efficiency means that further improvement of one of the planning goals such as high conformity, steep dose gradient, or low treatment time can only be achieved at the expense of one of the other goals. The DAP is an established concept in diagnostic radiology, where it is used to estimate the dose exposure of patients during X-ray examinations or interventions on the basis of the “amount of radiation” emitted by the X-ray unit, which is measured using a large-area transmission ionization chamber dosimeter [10], [11]. DAPs have also been applied in small field dosimetry and quality assurance of photon beams [12], [13], [14], [15], [16], [17], [18], [19], [20], [21], [22]. However, this concept is not used in the therapeutic application of X-rays, where the dose distribution in the patient resulting from a given radiation field configuration can be calculated accurately by means of modern three-dimensional treatment planning methods based on volumetric CT images. The idea here was to investigate whether the DAP can be used as a measure for the efficiency of a radiation field configuration in covering a target volume by relating the calculated dose-area product of a beam set to the dose achieved in the target volume. The usefulness of the method was demonstrated in a retrospective planning data set.

## 2 Methods and materials

### 2.1 Theory

As a measure for the targeting efficiency of a beam set, DAP_ratio_, the integral DAP of the applied beam set, *DAP_beamset_*, was related to the DAP deposited in the target, *DAP_target_*,

(1) $${\text{DAP}}_{\text{ratio}}\text{=}\frac{{\mathrm{DAP}}_{\mathrm{beamset}}}{{\mathrm{DAP}}_{\mathrm{target}}}$$

The DAP deposited in the target, *DAP_target_*, was derived from the average dose *D_mean_* deposited in the target volume *V_target_* using

(2) $${\mathrm{DAP}}_{\mathrm{target}}\;\text{=}\;D_{\mathrm{mean}}∙A_{\mathrm{target}}=D_{\mathrm{mean}}∙\pi {\left(3V_{\mathrm{target}}/4\pi \right)}^{2/3}$$

and where *A_target_* was assumed to be the cross-section of a spherical volume.

The integral DAP of the beam set, *DAP_beamset_* was calculated following the ray-tracing dose calculation algorithm of the Cyberknife [23] by superposition of the DAP of all beams

(3) $${\mathrm{DAP}}_{\mathrm{beamset}}\text{=}\sum_{i\in beamset}{\mathrm{MU}}_{i}∙{\mathrm{OF}}_{i}∙{\mathrm{TPR}}_{i}∙{\left(\frac{800}{{\mathrm{SAD}}_{i}}\right)}^{2}∙\underset{100\%}{\int^{50\%}}{\mathrm{OCR}}_{i}∙R\text{d}R\text{d}\varphi$$

Here *MU* is the number of monitor units, *OF* the output factor, *TPR* the tissue-phantom ratio, *SAD* the source-axis distance and *OCR* the off-center ratio. Note that in the calculation of DAPs, we restricted the integration over radius *R* and azimuth angle φ to the in-field region of the beams with *OCR(R,* φ*)* ≥ 50%. By this definition, the DAP_ratio_ is less than 1 when the in-field regions of the beams fully contribute to the dose in the target, while the DAP_ratio_ is greater than 1 if the in-field dose of the beams is also distributed outside the target. Thus, DAP values above 1 indicate a suboptimal treatment plan with avoidable dose exposure to normal tissue.

### 2.2 Treatment planning study

In order to demonstrate the usefulness of DAP_ratio_ to determine Pareto efficiency of a treatment plan with respect to collimator selection we selected 5 clinical targets representative for the typical range of volumes of singular brain metastases treated with the Cyberknife (Supplementary Materials, S1). Target volumes were 0.50, 0.92, 2.01, 3.07 and 5.68 ml. In all cases, a margin of zero was applied so that the planning target volume PTV is equal to the clinical target volume CTV. For each target, a variety of plans were calculated using the same optimization goals and weights as used for the applied clinical case, while varying the collimator setting. All collimators with sizes smaller than the equivalent diameter of the target and all combinations of 2 or 3 of these collimators were used. This resulted in up to 41 different collimator settings per target and a total of 121 plans for all targets. The optimization script included minimum and maximum dose as target goals for the PTV. Several shell structures were generated and used as critical goals for the optimization of the conformity and dose gradient, as well as to minimize the dose to healthy brain tissue. Relevant organs at risk (brainstem and the optical system) are added separately as critical goals (detailed planning parameters in the Supplementary Materials, S2). For all plans, the prescribed dose was 20 Gy at the 65% (±10%) isodose level, and the intended coverage of 99.5–99.8% was adjusted to the value achieved in the clinical plan. Following ICRU recommendations and standard clinical practice, coverage was in all cases >99% [1], [9]. The Cyberknife planning software Precision 2.0.1.1 (Accuray, Sunnyvale, CA, USA) and the VOLO^TM^ optimizer were used.

### 2.3 Data analysis and statistics

The influence of collimator selection on plan quality was analyzed in terms of conformity and dose gradient using the conformity index CI and the gradient index R50% and in terms of treatment time and total number of monitor units (TotalMU). The conformity index CI was defined as $\text{CI}\;={\left({\mathrm{PTV}}_{\mathrm{PIV}}\right)}^{2}/\left(PTV∙PIV\right)$, where *PTV* is the planning target volume, *PIV* the prescription isodose volume, and *PTV_PIV_* the planning target volume that is covered by the prescription isodose volume [24], [25]. The gradient index R50% was defined as $\text{R50\textbackslash{}\%}\;=V_{PIV50\%}/PTV$, where *V_PIV_*_50_*_%_* is the volume that is encompassed by half of the prescription isodose [9], [26].

In addition, the DAP_ratio_ was compared with two recently proposed indices of plan quality, f12 and η50%. Here, f12 is a measure of how closely the optimal dose gradient is achieved and is defined as the ratio between the off-target volume of the 12 Gy isodose (V12Gy), a predictor of radionecrosis [27], and the lowest achievable V12Gy for a given tumor size and prescription isodose [28]. The energy efficiency index η50%, recently proposed by Dimitriadis and Paddick [29], combines conformity, dose gradient, and mean target dose into a single value by relating the integral dose received in the target volume to the integral dose received in the volume covered by 50% of the prescribed isodose.

Data analysis was performed using box plots and scatter plots. The statistical correlation of DAP_ratio_ with CI, R50%, treatment time, TotalMU, f12 and η50% was evaluated using Spearman's rank correlation coefficient ρ. Absolute values of ρ above 0.9 were defined as very strong correlation, values between 0.70–0.89 as strong correlation, values between 0.40–0.69 as moderate correlation, values between 0.10–0.39 as weak correlation and values under 0.1 were defined as negligible correlation [30]. The Kruskal-Wallis rank sum test was used to assess the differences in DAP_ratio_ for each target and for the number of collimators. The F-test was used to compare the variances in DAP_ratio_ for plans with different number of collimators. The significance level p = 0.05 was used. The results were expressed as mean ± standard deviation. Statistical evaluation was performed with R v3.6.3 (https://www.r-project.org).

## 3 Results

**Figure 1:**
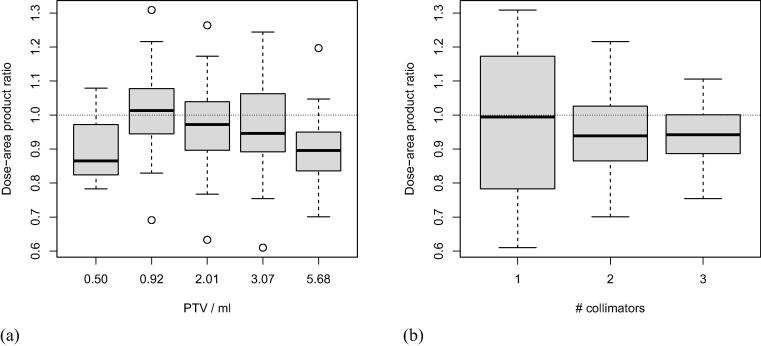
Box plot of DAP_ratio_ as a function of (a) planning target volume and (b) number of collimators.

The DAP_ratio_ for all plans averaged 0.95 ± 0.13 (median: 0.94, range: 0.61–1.31) and, despite significant differences (p = 0.007), showed a similar variance for all targets (Fig. 1a). The DAP_ratio_ of the plans showed a marked dependence on the number of collimators used (Fig. 1b). While the average DAP_ratio_ was independent of the number of collimators (0.97, 0.95, and 0.94 for plans with 1, 2, and 3 collimators, respectively; p = 0.80), the corresponding variances differed significantly (±0.21, ±0.12, and ±0.08 for plans with 1, 2, and 3 collimators, respectively; p = 2.2·10^−16^). The DAP_ratio_ was greater than 1 in 50% (11/22) of plans with one collimator, in 35% (16/46) of plans with two collimators, and in 26% (14/53) of plans with three collimators.

**Figure 2:**
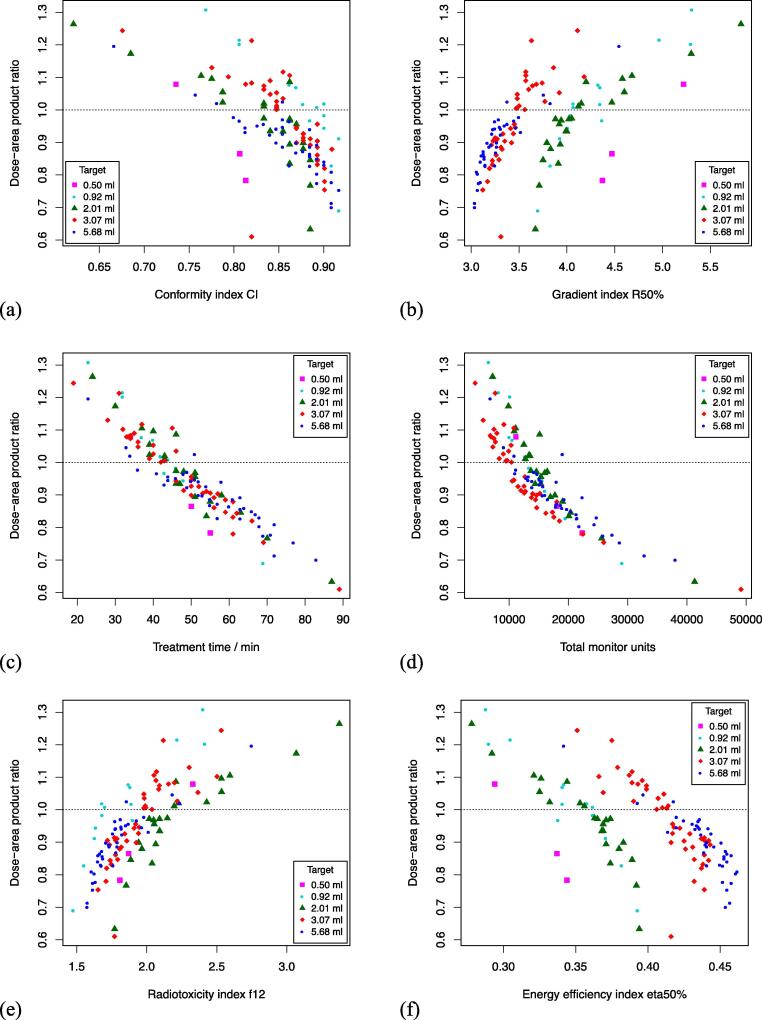
Scatter plot of DAP_ratio_ as a function of (a) conformity index CI, (b) gradient index R50%, (c) treatment time, (d) total monitor units, (e) radiotoxicity index f12, and (f) of energy efficiency index η50%.

Fig. 2 shows scatter plots demonstrating the strong relationship between DAPratio and CI, R50%, treatment time, TotalMU, f12, and η50%. For each target, DAP_ratio_ was strongly or very strongly correlated with all other metrics of plan quality (Table 1). Only for R50% and η50% was there a moderate correlation with DAP_ratio_ when the plans of all targets were combined for analysis.

**Table 1:** Spearman correlation coefficients ρ of DAP_ratio_ and conformity index CI, gradient index R50%, treatment time, total monitor units (TotalMU), radiotoxicity index f12, and energy efficiency index η50% for all planning target volumes (PTV).

	PTV (ml)					
DAP_ratio_ ∼	all	0.50	0.92	2.01	3.07	5.68
CI	−0.711	−1.000	−0.929	−0.875	−0.805	−0.877
R50%	0.685	1.000	0.874	0.944	0.912	0.900
Treat.Time	−0.944	−1.000	−0.985	−0.938	−0.958	−0.934
TotalMU	−0.895	−1.000	−0.982	−0.936	−0.937	−0.907
f12	0.804	1.000	0.842	0.928	0.916	0.910
η50%	−0.673	−1.000	−0.877	−0.951	−0.870	−0.885

**Figure 3:**
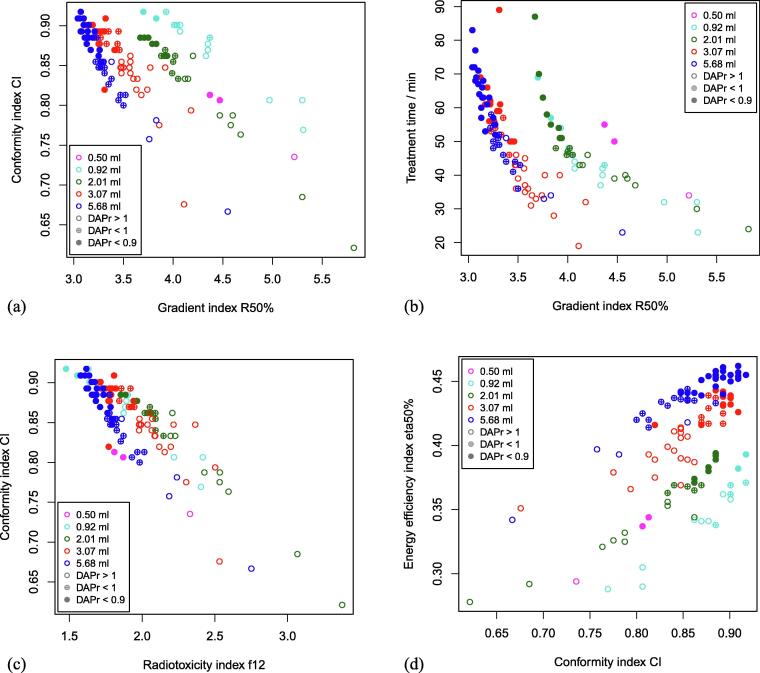
Scatter plots of (a) conformity index CI and gradient index R50%, (b) treatment time and gradient index R50%, (c) conformity index CI and radiotoxicity index f12, and (d) energy efficiency index η50% and conformity index CI. Points are shown as open circles when DAP_ratio_ was greater than 1, as crossed circles when DAP_ratio_ was between 0.9 and 1, and as closed circles for DAP_ratio_ less than 0.9.

The ability of DAP_ratio_ to identify Pareto-efficient treatment plans is demonstrated in Fig. 3. Plans with high conformity (high CI) and high dose gradient (low R50% or low f12) were clearly associated with DAP_ratio_ < 1, and plans with very high conformity and very high dose gradient were associated with DAP_ratio_ < 0.9 (Fig. 3a,c). Similar results were obtained for plans in relation to CI and η50% (Fig. 3d). The trade-off between plan quality and treatment time is clearly evident in Fig. 3b. Treatment plans with a steep dose gradient (low R50%) were strongly correlated with a long treatment time and are associated with a low DAP_ratio_. For plans with treatment times less than 30 minutes, the DAP_ratio_ was greater than 1.13, whereas for plans with treatment times greater than 60 minutes, it was less than 0.893.

## 4 Discussion

In this study, we developed a method for determining Pareto-efficient treatment plans with respect to collimator selection in robotic SRS. The concept of DAP was used to define a measure of the targeting efficiency of a beam set, the DAP_ratio_, by relating the integral DAP of a beam set to the DAP deposited in the target. With this definition, a DAP_ratio_ of less than 1 indicates high targeting efficiency, where the in-field dose of the beams is focused to the target, while a plan with a DAP_ratio_ greater than 1 indicates suboptimal targeting efficiency, where the in-field dose is also distributed off-target. Thus, a Pareto-efficient plan is characterized by a value for DAP_ratio_ of less than 1.

In a clinical dataset, we were able to show that DAP_ratio_ was indeed distributed around 1, regardless of the target size and the number of collimators, and only the variance of the DAP_ratio_ decreased with increasing number of collimators. Moreover, our results show that DAP_ratio_ was strongly or even very strongly correlated with all other metrics of plan quality commonly used in SRS. Only when the plans of all targets were combined, we found a moderate correlation with R50% and η50%, as these two metrics exhibited a marked dependence on the size of the target volume. Taken together, this justifies the interpretation of DAP_ratio_ as a combined and uniformly scaled metric to characterize plan quality in robotic SRS that can be used to identify Pareto efficiency of a plan. In fact, plans with DAP_ratio_ < 1 generally showed superior plan quality in terms of dose conformity, dose gradient, and energy efficiency than plans with DAP_ratio_ > 1. As the DAP_ratio_ dropped below 1 to 0.9 or less, the quality of the plan continued to improve, but usually at the expense of longer treatment time and a higher total number of monitor units. We would like to emphasize, however, that our goal was not to overcome established plan metrics such as CI and R50%, but rather to complement them by introducing a measure of the Pareto efficiency of a plan with respect to collimator selection.

Like the energy efficiency index η50%, the DAP_ratio_ combines dose conformity and gradient in a single number. But unlike η50%, DAP_ratio_ is uniformly scaled and independent of target size. But like η50%, which by definition increases strongly as the prescribed isodose level decreases [29], DAP_ratio_ depends on dose homogeneity of the plan, due to normalization to the mean dose in the target volume (Equation (2)). Therefore, DAP_ratio_ should be used primarily to compare plans with similar prescribed isodose levels. Perhaps the dependence of DAP_ratio_ on dose homogeneity could be compensated for in a future version by using a variable integration limit (*OCR(R,* φ*)* ≥ x%) in Equation (3) adjusted to the prescribed isodose level.

Furthermore it should be noted that DAP_ratio_ depends on measured off-center ratios (Equation (3)) which are device dependent while η50% is calculated from dose-volume histograms and thus is independent of the treatment modality. Therefore, DAP_ratio_ may be of limited use for comparing plans between different devices and is primarily intended for comparing different plans using the same treatment modality. This is the case when DAP_ratio_ is used to determine the Pareto efficiency with respect to collimator selection, and thus it appears to be more suitable for this purpose due to its uniform scaling.

Another limitation of DAP_ratio_ may arise from the assumption of a spherical shape of the target volume (Equation (2)). This is justified for brain metastases that are by far the most common target in SRS but may be an issue in other targets enforcing some refinement in the definition of the denominator of DAP_ratio_. Furthermore, the proposed concept should also be extendable for use in multiple targets. It would also be interesting to investigate whether a similar concept could be used for collimators other than fixed collimators, such as the Iris or multileaf collimator [31], [32], [33].

Finally, we did not explicitly examine the impact of target coverage on DAP_ratio_. This is justified because in SRS, high coverage is the primary goal of treatment planning, typically 98% or higher [1], [9].

## 5 Conclusion

Based on the DAP, it was possible to define a measure of plan quality that describes the target efficiency of a set of beams by a single, uniformly scaled number. With this metric, the Pareto efficiency of a plan in robotic SRS with respect to collimator selection is indicated by values below 1, and values below 0.9 indicate the highest plan quality, but mainly at the expense of treatment time.

## Ethics statement

This retrospective study was approved by the local ethics committee of the University Hospital of Cologne (file number 16-476).

## Funding

The authors state that this work has not received any funding.

## Declaration of Competing Interest

The authors declare that they have no known competing financial interests or personal relationships that could have appeared to influence the work reported in this paper.

## References

[1] Seuntjens J., Lartigau E.F., Cora S., Ding G.X., Goetsch S., Nuyttens J. (2014). ICRU report 91. Prescribing, recording, and reporting of stereotactic treatments with small photon beams. J ICRU.

[2] Benedict S.H., Yenice K.M., Followill D., Galvin J.M., Hinson W., Kavanagh B. (2010). Stereotactic body radiation therapy: the report of AAPM Task Group 101. Med Phys.

[3] Adler J.R., Chang S.D., Murphy M.J., Doty J., Geis P., Hancock S.L. (1997). The Cyberknife: a frameless robotic system for radiosurgery. Stereotact Funct Neurosurg.

[4] Antypas C., Pantelis E. (2008). Performance evaluation of a CyberKnife G4 image-guided robotic stereotactic radiosurgery system. Phys Med Biol.

[5] Schweikard A., Schlaefer A., Adler J.R. (2006). Resampling: an optimization method for inverse planning in robotic radiosurgery. Med Phys.

[6] Schlaefer A., Schweikard A. (2008). Stepwise multi-criteria optimization for robotic radiosurgery. Med Phys.

[7] Zeverino M., Marguet M., Zulliger C., Durham A., Jumeau R., Herrera F. (2019). Novel inverse planning optimization algorithm for robotic radiosurgery: First clinical implementation and dosimetric evaluation. Phys Med.

[8] Hellerbach A., Eichner M., Rueß D., Luyken K., Hoevels M., Judge M. (2022). Impact of prescription isodose level and collimator selection on dose homogeneity and plan quality in robotic radiosurgery. Strahlenther Onkol.

[9] Eaton D.J., Lee J., Patel R., Millin A.E., Paddick I., Walker C. (2018). Stereotactic radiosurgery for benign brain tumors: Results of multicenter benchmark planning studies. Pract Radiat Oncol.

[10] Le Heron J.C. (1992). Estimation of effective dose to the patient during medical x-ray examinations from measurements of the dose-area product. Phys Med Biol.

[11] Zoetelief J., Dance D.R., Drexler G., Järvinen H., Rosenstein M. (2005). ICRU Report 74. Patient dosimetry for X rays used in medical imaging. J ICRU.

[12] Djouguela A., Harder D., Kollhoff R., Rühmann A., Willborn K.C., Poppe B. (2006). The dose-area product, a new parameter for the dosimetry of narrow photon beams. Z Med Phys.

[13] Dufreneix S., Ostrowsky A., Rapp B., Daures J., Bordy J.M. (2016). Accuracy of a dose-area product compared to an absorbed dose to water at a point in a 2 cm diameter field. Med Phys.

[14] Heidorn S.C., Kremer N., Fürweger C. (2016). A Novel Method for Quality Assurance of the Cyberknife Iris Variable Aperture Collimator. Cureus.

[15] Jurczak J., Rapp B., Delaunay F., Gouriou J., Dufreneix S., Bordy J.M. (2022). Dose area product primary standards established by graphite calorimetry at the LNE-LNHB for small radiation fields in radiotherapy. Phys Med.

[16] Kupfer T., Lehmann J., Butler D.J., Ramanathan G., Bailey T.E., Franich R.D. (2017). Commissioning of a PTW 34070 large-area plane-parallel ionization chamber for small field megavoltage photon dosimetry. J Appl Clin Med Phys.

[17] Niemelä J., Partanen M., Ojala J., Kapanen M., Keyriläinen J. (2021). Dose-area product ratio in external small-beam radiotherapy: beam shape, size and energy dependencies in clinical photon beams. Biomed Phys Eng Express.

[18] Niemelä J., Partanen M., Ojala J., Sipilä P., Björkqvist M., Kapanen M. (2017). Measurement and properties of the dose-area product ratio in external small-beam radiotherapy. Phys Med Biol.

[19] Pimpinella M., Caporali C., Guerra A.S., Silvi L., De Coste V., Petrucci A. (2018). Feasibility of using a dose-area product ratio as beam quality specifier for photon beams with small field sizes. Phys Med.

[20] Poppe B., Thieke C., Beyer D., Kollhoff R., Djouguela A., Rühmann A. (2006). DAVID–a translucent multi-wire transmission ionization chamber for in vivo verification of IMRT and conformal irradiation techniques. Phys Med Biol.

[21] Razinskas G., Wegener S., Greber J., Sauer O.A. (2018). Sensitivity of the IQM transmission detector to errors of VMAT plans. Med Phys.

[22] Underwood T.S., Winter H.C., Hill M.A., Fenwick J.D. (2013). Detector density and small field dosimetry: integral versus point dose measurement schemes. Med Phys.

[23] Accuray Incorporated (2018). Physics Essentials Guide. CyberKnife® Treatment Delivery System. 1059805-ENG A. Version 11.1.x. Date of Revision.

[24] van't Riet A., Mak A.C., Moerland M.A., Elders L.H., van der Zee W. (1997). A conformation number to quantify the degree of conformality in brachytherapy and external beam irradiation: application to the prostate. Int J Radiat Oncol Biol Phys.

[25] Paddick I. (2000). A simple scoring ratio to index the conformity of radiosurgical treatment plans. Technical note J Neurosurg.

[26] Timmerman R., Paulus R., Galvin J., Michalski J., Straube W., Bradley J. (2010). Stereotactic body radiation therapy for inoperable early stage lung cancer. JAMA.

[27] Lawrence Y.R., Li X.A., el Naqa I., Hahn C.A., Marks L.B., Merchant T.E. (2010). Radiation dose-volume effects in the brain. Int J Radiat Oncol Biol Phys.

[28] Hellerbach A., Luyken K., Hoevels M., Gierich A., Rueß D., Baus W.W. (2017). Radiotoxicity in robotic radiosurgery: proposing a new quality index for optimizing the treatment planning of brain metastases. Radiat Oncol.

[29] Dimitriadis A., Paddick I. (2018). A novel index for assessing treatment plan quality in stereotactic radiosurgery. J Neurosurg.

[30] Schober P., Boer C., Schwarte L.A. (2018). Correlation Coefficients: Appropriate Use and Interpretation. Anesth Analg.

[31] Echner G.G., Kilby W., Lee M., Earnst E., Sayeh S., Schlaefer A., Rhein B., Dooley J.R., Lang C., Blanck O., Lessard E., Maurer C.R., Schlegel W. (2009). The design, physical properties and clinical utility of an iris collimator for robotic radiosurgery. Phys Med Biol.

[32] Asmerom G., Bourne D., Chappelow J., Goggin L.M., Heitz R., Jordan P., Kilby W., Laing T., Maurer C.R., Noll J.M., Sayeh S., Weber A. (2016). The design and physical characterization of a multileaf collimator for robotic radiosurgery. Biomed Phys Eng Express.

[33] Fürweger C., Prins P., Coskan H., Heijmen B.J. (2016). Characteristics and performance of the first commercial multileaf collimator for a robotic radiosurgery system. Med Phys.

