# Supplementary material for: Use of dose-area product to assess plan quality in robotic radiosurgery
Source: Z Med Phys. 2023 Jan 28;34(3):428–35. doi: 10.1016/j.zemedi.2023.01.001 (PMC11384082; doi:10.1016/j.zemedi.2023.01.001)
Supplement: Supplementary Data 1 [file mmc1.docx]

**Supplementary materials**

**S1 Patient data**

Figure S 1 Treatment plans of five patients treated with the Cyberknife for a single brain metastasis with a volume of (a) 0.50 ml, (b) 0.92 ml, (c) 2.01 ml, (d) 3.07 ml, and (e) 5.68 ml.

**S2 Treatment planning**

Dose calculations were performed using the Ray-Tracing algorithm and a high-resolution computational grid encompassing the entire skull. Fixed collimators were selected and the treatment anatomy was set to head_iris-fixed, using the Full_Path with a maximum number of 133 available nodes.

A target boundary distance of 0 was always used when selecting collimators and assembling collimator sets.

In all cases, the iterations per optimization was 50, the maximum number of beams was 300, and the minimum MU per beam was 10.

For the respective PTV, a minimum dose of 20.00 Gy and a maximum dose of 30.77 Gy were set as target goals. The optimization script included multiple shell structures (Table S1) and relevant organs at risk with corresponding clinical dose limits as critical goals. Optimization weights from 1 to 10 were used to account for the respective dose constraints of the 5 different clinical cases.

Eyes were blocked to beam crossings, chiasm and optic nerves were set to exit only in all cases.

Table S 1 Planning parameters used for the five planning target volumes (PTV).

| **PTV [ml]** | **MU-penalty** | **Max-MU/Beam** | **Shells** | **Shell size [mm]** | **Organs at risk** |
| --- | --- | --- | --- | --- | --- |
| 0,50 | 0,7 | 150 | 9 | 2/5/10/15/20/30/50/70/100 | brain |
| 0,92 | 0,7 | 150 | 7 | 2/7/12/20/30/50/70 | brain |
| 2,01 | 0,7 | 120 | 9 | 2/7/10/15/20/30/50/70/100 | brain, chiasm, brain stem |
| 3,07 | 0,7 | 150 | 6 | 2/7/12/20/40//70 | brain |
| 5,68 | 0,5 | 150 | 9 | 2/5/10/15/20/30/50/70/100 | brain |
